# Germinative and Post-Germinative Behaviours of *Brassica napus* Seeds Are Impacted by the Severity of S Limitation Applied to the Parent Plants

**DOI:** 10.3390/plants8010012

**Published:** 2019-01-05

**Authors:** Philippe D’Hooghe, Dimitri Picot, Sophie Brunel-Muguet, Stanislav Kopriva, Jean-Christophe Avice, Jacques Trouverie

**Affiliations:** 1 Normandie Univ, UNICAEN, INRA, UMR EVA, SFR Normandie Végétal FED4277, Esplanade de la Paix, F-14032 Caen, France; philippe.dhooghe@live.fr (P.D.); dimi.picot@gmail.com (D.P.); sophie.brunel-muguet@unicaen.fr (S.B.-M.); jean-christophe.avice@unicaen.fr (J.-C.A.); 2 Botanical Institute and Cluster of Excellence on Plant Sciences (CEPLAS), University of Cologne, 50674 Cologne, Germany; skopriva@uni-koeln.de

**Keywords:** S metabolism, ^34^S labelling, sulphate uptake, sulphate translocation, seed viability, oilseed rape

## Abstract

In oilseed rape (*Brassica napus* L.), sulphur (S) limitation leads to a reduction of seed yield and nutritional quality, but also to a reduction of seed viability and vigour. S metabolism is known to be involved in the control of germination sensu stricto and seedling establishment. Nevertheless, how the germination and the first steps of plant growth are impacted in seeds produced by plants subjected to various sulphate limitations remains largely unknown. Therefore, this study aimed at determining the impact of various S-limited conditions applied to the mother plants on the germination indexes and the rate of viable seedlings in a spring oilseed rape cultivar (cv. Yudal). Using a ^34^S-sulphate pulse method, the sulphate uptake capacity during the seedling development was also investigated. The rate of viable seedlings was significantly reduced for seeds produced under the strongest S-limited conditions. This is related to a reduction of germination vigour and to perturbations of post-germinative events. Compared to green seedlings obtained from seeds produced by well-S-supplied plants, the viable seedlings coming from seeds harvested on plants subjected to severe S-limitation treatment showed nonetheless a higher dry biomass and were able to enhance the sulphate uptake by roots and the S translocation to shoots.

## 1. Introduction

Oilseed rape (*Brassica napus* L.) is particularly sensitive to sulphur (S) limitation that results in reduced yield and seed quality. This lack of seed quality may have unexpected impacts on seed germination and/or on the viability of subsequent seedlings. Indeed, mature seeds produced by oilseed rape subjected to a sulphate restriction applied early in the cycle of development (i.e., at the bolting stage), were characterised by a low ability to produce normal seedlings [1] and by reduced germination vigor and viability [2]. Seed germination sensu stricto is a two-phase process that starts with the uptake of water by the quiescent dry seed and ends with the elongation of the embryonic axis. The completion of germination is visible by the penetration of the structures surrounding the embryo by the radicle (radicle protrusion), and subsequent events are more specifically associated with seedling establishment [3,4,5]. Germination sensu stricto and seedling establishment are complex processes that possess multiple and variable levels of regulation that depend on the studied species [1,3,5,6,7]. In oilseed rape, mature seeds are non-dormant, but can enter into secondary dormancy if environmental conditions are not favourable for germination [8]. From this late growth stage, the sulphate previously stored by the plant and actively remobilised to seeds can meet the needs in S for a good seed development and quality [1,2]. However, if the sulphate shortage occurs earlier in plant development, S reserves are not sufficient to satisfy the needs for plant growth and seed development, and the mature seeds produced can display reduced accumulation of S and lipid reserves and profound metabolic changes that impact seed viability and do not favour seed germination of the still-viable seeds [2].

Sulphur metabolism is involved at various levels in the control of germination sensu stricto and seedling establishment [7]. For example, S-adenosylmethionine (SAM), synthesised by SAM synthetase from methionine, is a methyl-donor group but also a precursor of biotin and ethylene and thus could interplay at multiple levels in the regulation of these key steps [7,9,10,11]. Sulphur limitation is well-known for modulating the S management in Brassicacea species by increasing the sulphate uptake and remobilisation through the induction of the corresponding transporters (e.g., the *BnSultr1* and *BnSultr4* genes for root uptake and remobilisation of sulphate stored in a vacuole, respectively) and by increasing assimilation capacities [12,13]. S restriction could also cause a profound disturbance in seed metabolism, reduce the accumulation of seed reserves and reduce seed viability and germination vigour [2]. According to our knowledge, no study has sufficiently considered the development and the modulations of sulphate management of seedlings generated from seeds harvested of S-limited plants. In order to investigate these processes, we performed experiments using seeds harvested from plants subjected to various S-limitations during their growth. Germination indexes (such as speed of germination, time to reach 50% of germinated seeds, germination capacity) and the rate of normal seedlings produced were determined. The seeds were used (i) to verify if the germination and the subsequent seedlings’ establishment were affected and (ii) to evaluate the sulphate uptake capacity of the green seedlings developed from these seeds by using a specific ^34^S-sulphate pulse method.

## 2. Results

### 2.1. Impacts of Suboptimal Levels of Sulphate Applied from the Beginning of the Plant Growth Cycle on S Status of Seeds and Its Consequence for Germination

The S400%, S70%, S20% and S5% seeds correspond to seeds harvested from plants supplied with 400%, 70%, 20% and 5%, respectively, of the optimal level of sulphate they need for growth. The mature seed dry weights and the amounts of C and N were not significantly different between the four seed lots. Compared to S400% seeds, the S70% seeds had similar S, S-sulphate and S-glutathione amounts but a higher S-protein amount (Table 1). The S20% seeds showed lower S and S-sulphate amounts than S400% seeds. Similarly, S5% seeds showed reduced S and S-sulphate amounts, and a reduced S-glutathione amount, compared to S400% seeds (Table 1).

**Figure 1:**
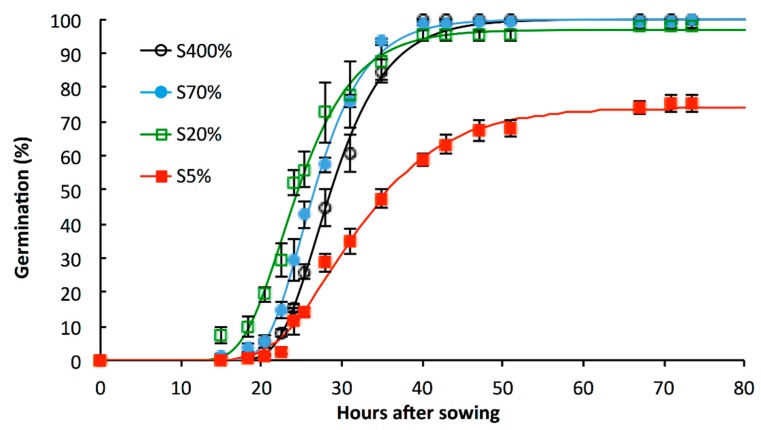
Evolution of germination percentage of *Brassica napus* seeds obtained from plants well S supplied and grown under S-limited conditions. Values shown by symbols correspond to means ± SE (n = 3). Seeds were obtained from plants subjected to plethoric S nutrition (400% of plant S needs provided, S400% seeds) or subjected to a S limitation from the beginning of plant growth with 70%, 20% and 5% of plant S needs provided (S70%, S20% and S5% seeds, respectively). The curves are calculated from the non-linear Gompertz regression model [14]. The germination indexes determined from these observations are given in Table 2.

The germination of S400% seeds began around 20 h after sowing and reached its maximum around 50 h after sowing (Figure 1). Compared to S400% seeds, germination was not affected for S70% and S20% seeds. However, S5% seeds showed a lower germination capacity (by 24.7%) and speed of germination (by 32.6%) than S400% seeds (Table 2). While the time to reach 50% of the germination capacity (T’50) was not significantly different from S400% seeds, the time to reach 50% of germination (T50) calculated for S5% seeds was significantly higher.

### 2.2. Development and S Management of Green Seedlings

**Figure 2:**
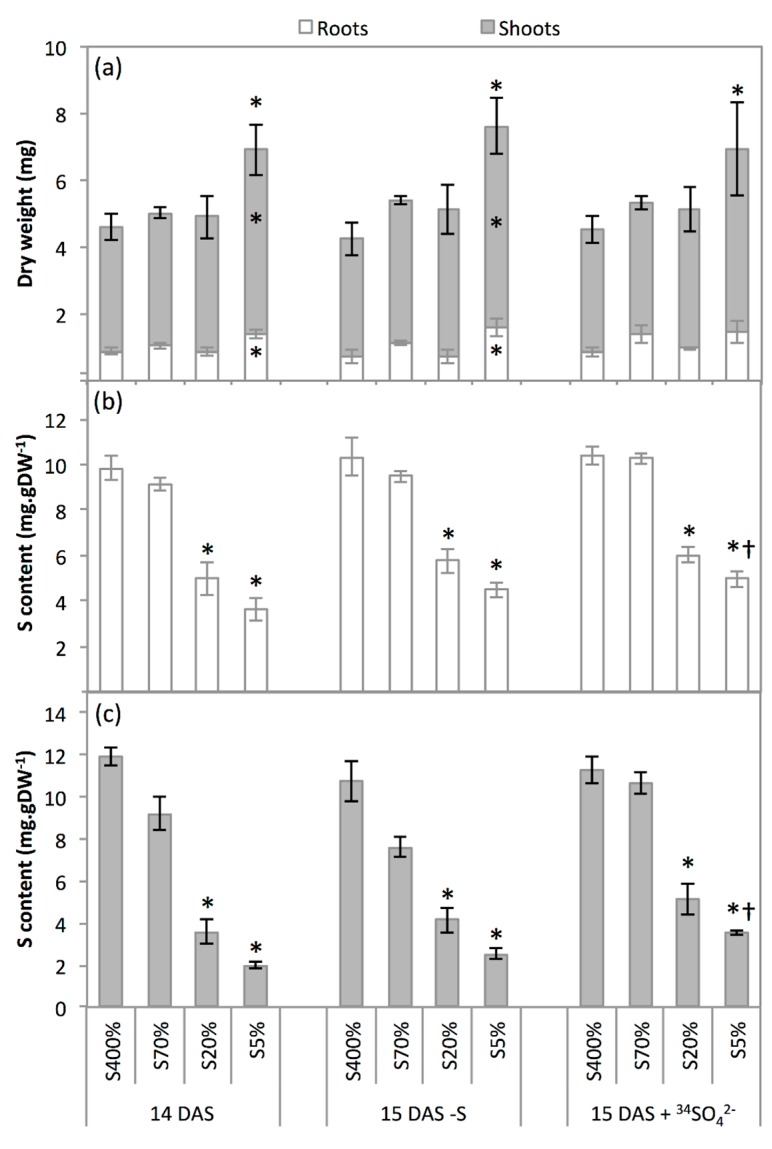
Dry weight and S content of green seedlings shoots and roots. (**a**) Dry weight of green seedlings shoots and roots. (**b**) S content of green seedlings roots. (**c**) S content of green seedlings shoots. Data were acquired 14 days after sowing on water (14 DAS) and after 24-h treatment of 14 DAS seedlings with 508.7 µM ^34^SO_4_^2−^ at 5 atom% excess (15 DAS + ^34^SO_4_^2−^) and with 8.7 µM SO_4_^2−^ (15 DAS-S). Seedlings were grown from seeds harvested on plants subjected to plethoric S nutrition (400% of plant S needs provided, S400% seeds) or subjected to a S limitation from the beginning of plant growth with 70%, 20% and 5% of plant S needs provided (S70%, S20% and S5% seeds, respectively). Values correspond to means ± SE (n = 3). * Significant difference from the value of S400% green seedlings (*p* < 0.05). † Significant difference from the corresponding value at 14 DAS (*p* <0.05). In panel (**a**), signs (* and/or †) on the top of the bars are for the whole plant (shoot and root), while signs inside the bar are for root or shoot specifically.

The normal seedling establishment rate was significantly reduced for S70%, S20% and S5% seeds compared to S400% seeds (Table 2). Fourteen days after sowing on perlite (14 DAS), the green seedlings from S400%, S70% and S20% seeds weighted about 5 mg dry weight, with a shoot:root ratio around 4. Interestingly, the S5% green seedlings had a significantly higher dry weight in both shoots and roots (Figure 2a). The shoot:root ratio was not significantly different between S400% and S5% green seedlings. The S amounts in seedlings produced from all of the seed lots (Supplemental Figure S1a) were not different to the S amounts measured in mature seeds (Table 1), showing that no S was absorbed during the 14 days of seedling establishment.

In order to minimise the differences linked to variations in biomass between S400%, S70%, S20% and S5% green seedlings, but also between 14 and 15 DAS, the results were expressed as contents. However, the expression of results in terms of quantity (see Supplemental Figures S1 and S2) reflects the same observations and does not change the interpretations. The S contents were similar between shoot and root for S400% and S70% green seedlings. These contents were significantly lower in S20% and S5% green seedlings, particularly for the shoot (Figure 2b,c). At 14 DAS, the S was principally allocated to the shoot, with 83% of the total S in S400% green seedlings; however, this preferential allocation to the aerial parts tended to be reduced in S70%, S20% and S5% green seedlings (with up to 32% of the total S in the root for S5% green seedlings; Supplemental Figure S1b).

**Figure 3:**
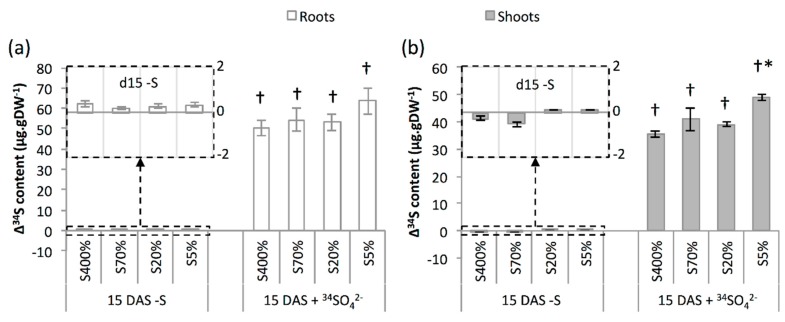
Differences in ^34^S (Δ^34^S) content in excess in roots (**a**) and shoots (**b**) of oilseed rape green seedlings between 14 DAS and 15 DAS-S and between 14 DAS and 15 DAS + ^34^SO_4_^2−^. Values correspond to means ± SE (n = 3). Seedlings were grown from seeds harvested on plants subjected to plethoric S nutrition (400% of plant S needs provided, S400% seeds) or subjected to a S limitation from the beginning of plant growth with 70%, 20% and 5% of plant S needs provided (S70%, S20% and S5% seeds, respectively). † Significant difference from the 15 DAS-S value (*p* <0.05). * Significant difference from the corresponding value of S400% seedlings (*p* < 0.05).

The feeding of 14 DAS green seedlings from S400%, S70%, S20% and S5% seeds for 24 h with the nutrient solution containing ^34^S-sulphate (5 atom% excess; 15 DAS + ^34^SO_4_^2−^) or containing no S (15 DAS-S) did not change significantly the roots and shoot dry weights (Figure 2a). The S contents in the shoot and roots were not significantly affected by the -S treatment for all of the lots (Figure 2b,c). The supply with sulphate for 24 h (^+34^SO_4_^2−^) increased the S content in roots and shoots of S5% green seedlings, but did not change the S contents in roots and shoots for S400%, S70% and S20% green seedlings (Figure 2b,c). As expected, no significant difference in ^34^S contents in root and shoot was observed between 14 and 15 DAS in response to the -S treatment, and the ^+34^SO_4_^2−^ treatment increased markedly the ^34^S content in both shoot and root of all the green seedlings lots (Figure 3a,b). In terms of quantity, the ^34^S absorbed between 14 and 15 DAS was principally allocated to shoots (Supplemental Figure S2). Interestingly, the difference in ^34^S content between 15 DAS + ^34^SO_4_^2−^ and 14 DAS was higher in shoots of S5% green seedlings than in S400% green seedlings (Figure 3b).

**Figure 4:**
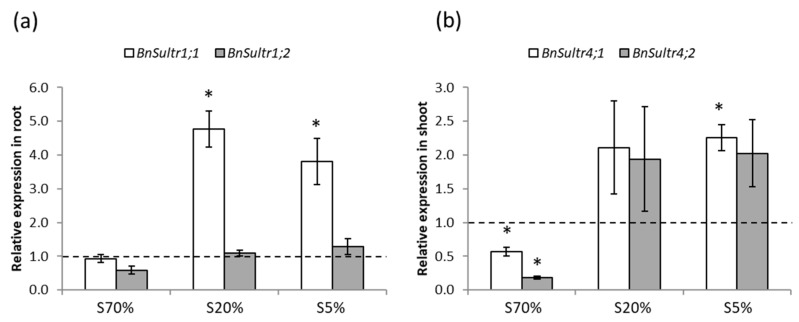
Relative expression of *BnSultr1;1* and *BnSultr1;2* in roots (**a**) and *BnSultr4;1* and *BnSultr4;2* in shoots (**b**) of S70%, S20% and S5% oilseed rape green seedlings 14 days after sowing on water (14 DAS). The expression level of *BnSultr1;1*, *BnSultr1;2*, *BnSultr4;1* and *BnSultr4;2* is relative to the result obtained in S400% green seedlings at 14 DAS, shown by the dotted line. Seedlings were grown from seeds harvested on plants subjected to plethoric S nutrition (400% of plant S needs provided, S400% seeds) or subjected to a S limitation from the beginning of plant growth with 70%, 20% and 5% of plant S needs provided (S70%, S20% and S5% seeds, respectively). Values correspond to means ± SE (n = 3) (*p* < 0.05). * Significant difference from the value of S400% green seedlings (*p* < 0.05).

To test whether the difference in ^34^S content is caused by different regulation of sulphate transporters, the transcript levels of genes encoding major sulphate transporters were compared by qPCR. As compared to S400% green seedlings at 14 DAS, no significant difference was observed for *BnSultr1;2* expression in roots of S70%, S20% and S5% green seedlings (Figure 4a) and for *BnSultr4;2* expression in shoots of S20% and S5% green seedlings (Figure 4b). *BnSultr1;1* gene expression was higher in roots of S20% and S5% green seedlings, and *BnSultr4;1* gene expression was higher in shoots of S5% green seedlings only. The expression of both *BnSultr4* genes was lower in shoots of S70% green seedlings compared to S400% seedlings (Figure 4).

## 3. Discussion

### 3.1. S-Limitation Impacts on Germination Sensu Stricto and on Post-Germinative Events

Under our experimental conditions, the germination kinetics (Figure 1) and the germination indexes (Table 2) of the seeds harvested on the spring genotype Yudal well-supplied with sulphate (i.e., S400% seeds) are not significantly different to the winter cv. Capitol used in our previous work [2]. The seeds produced by plants grown under severe S-limited conditions (i.e., S5% seeds) are characterised by a low viable seedlings establishment rate that is related to a reduction in germination vigour (Figure 1 and Table 2). The different germination indexes used in this study highlight that both speed of germination and germination capacity are reduced for the genotype studied in response to S5% treatment (Table 2). This observation is reinforced by similar findings in another oilseed rape genotype (cv. Capitol) and under a different severe S-limited condition applied to the mother plant [2] (i.e., a S restriction applied from the bolting stage [2] versus a severe S limitation from the beginning of growth in the present study). Interestingly, the low rate of viable seedlings for the S70% and S20% seeds was also associated with impacts on post-germinative events, since the normal seedling rates were significantly lower than the germination capacities.

The decline of germination capacity and vigor that is observed in S5% seeds could be related to various factors. First, the disturbances of C metabolism in developing and mature seeds in response to severe S limitation could reduce seed germination vigor [2]. Second, the modifications of synthesis, accumulation and perception of phytohormones in seeds with a low S status could be involved in the decrease of germination capacity and vigor. Among the candidates, ABA has an inhibitory role on testa rupture during seed germination of *Brassica napus* [16,17] and its accumulation is affected by sulphate starvation in *Arabidopsis thaliana* [18]. While a change in the ABA:GA balance to the benefit of ABA promotes dormancy, a modification of this balance in favour of GA promotes germination [6]. A sulphate restriction applied from the beginning of pollination did not impact ABA or GA contents significantly, but slightly increased the ABA:GA ratio without negative effect on germination vigour [19]. In addition, ethylene may also interact with ABA upon germination, counteracting its inhibitory effect in *Arabidospis thaliana* [20,21], and is involved in post-germinative events [22]. Under a S-limited condition, a lower availability of methionine could lead to a reduced production of SAM in seeds and subsequently to a decrease of the ethylene production that normally negatively impacts seed germination and seedling establishment. Therefore, the potential involvement of phytohormones on the seed germination capacity and vigor may deserve to be explored in a specific study in relationship with the level of S fertilization.

### 3.2. Seedling Sulphate Uptake Capacity Is Impacted by S Limitation Applied to the Parent Plant

The roots and shoots dry weights observed at 14 DAS for the S5% green seedlings were higher than for the S400% green seedlings (Figure 2a). These unexpected data highlight that seedling establishment could be strongly stimulated in seeds produced from plants severely limited in sulphate. Even though this enhanced biomass in offspring of plants submitted to S limitation is surprising, similar observations were made for *Brassica napus* in response to drought stress [23] and for *Arabidopsis thaliana* in response to salt stress [24]. Although it was not the matter of the present study, several hypotheses could be proposed to explain this positive transgenerational effect on seedling growth after germination completion in response to sulphate restriction, such as changes induced by stress in the epigenome of the seeds or maternal imprinting. Indeed, in addition to lipid and protein alterations [2], profound metabolic modifications may occur during seed development due to the lower S abundance, such as alteration of SAM, glutathione, acetyl-CoA or glucosinolate content [2,22,25,26] or other perturbations of homeostasis in seeds.

As seedlings were grown on water and under light for 14 DAS, an enhanced photosynthetic capacity of S5% green seedlings compared to S400% green seedlings could be a physiological process susceptible to explain the apparent discrepancy between similar seed and higher green seedling dry weights. Interestingly, this explanation is in agreement with previous hypothesis on maternal imprinting, since a lower SAM availability would impair ethylene production, which has been shown to be essential for achieving normal photosynthetic capacity in Arabidopsis leaves [27] and which is known to inhibit *Arabidopsis thaliana* root and shoot growth [28,29]. Moreover, due to its significant role in the modification of the epigenome through DNA methylation, SAM may also be potentially involved in the peculiar behaviour observed in S5% green seedlings.

Interestingly, as indicated by the higher S and ^34^S amounts and contents between 14 and 15 DAS (Figure 2b,c and Figure 3 and Supplemental Figures S1 and S2), the S5% green seedlings also showed a significantly higher sulphate uptake compared to S400% green seedlings. However, the role of the *BnSultr1;1* and *BnSultr1;2* genes’ expression in this higher sulphate uptake capacity cannot be clarified, since *BnSultr1;1* gene expression in the root was higher for S5% green seedlings, but also for S20% green seedlings (Figure 4), that showed no difference in ^34^S content compared to S400% green seedlings (Figure 3). Although this result might suggest a post-transcriptional regulation of this transporter, the involvement of other sulphate transporters in the higher sulphate uptake capacity of S5% green seedlings deserves to be studied. In addition to increasing the quantity and content of ^34^S more significantly in S5% green seedlings than in S400% green seedlings, the supply with 500 µM sulphate labelled with ^34^S (5 atom% excess) appears to change the allocation of S in S5% green seedlings in favour of the shoot. Consistent with this observation, the shoot of *Brassica oleracea* functioned as the primary sink for sulphate taken up after sulphate resupply to sulphate-deprived plants [30]. According to all of these results, it could be hypothesised that *BnSultr2;1*, a sulphate transporter responsible for the translocation of sulphate between roots and shoots and upregulated in roots under S-limiting conditions [31], is highly expressed in root of S5% green seedlings, explaining both their better sulphate uptake capacity and the translocation of the S taken up to the shoot.

This study highlights an induced-intergenerational effect of sulfur deficiency applied on mother plants on the uptake and translocation sulfur capacities of the offspring green seedlings. Seeds harvested on the most severe S-limited plants showed lower germination capacity; however, surprisingly, those that were able to produce normal seedlings displayed a higher growth. These behaviors raised questions about the S effect that could contribute to the stress memory of the offspring.

## 4. Materials and Methods

To address the objectives of the present work, the experiments were conducted on a spring oilseed rape genotype (cv. Yudal) with the aim to verify if the germination and the subsequent development of seedlings were affected in seeds that were obtained from plants supplied with different levels of sulphate.

### 4.1. Production of Seeds from Plants Supplied with Different Levels of Sulphate

The mature seeds from cv. Yudal were harvested on plants grown into individual pots (a mix of 2:3 perlite and 1:3 vermiculite) under greenhouse conditions (natural day/light conditions from January until July) and supplied with four different levels of S nutrition. Nutrient solutions were provided with an increasing amount of MgSO_4_ according to the relative-addition rate nutrient-dosing system to reach a constant relative growth rate during exponential plant growth as described by Brunel-Muguet et al. [32]. The four treatments were as follows: plants were supplied with 400% (i.e., plethoric S nutrition), 70%, 20% and 5% of the optimal S needs as sulphate from the beginning of the plant growth cycle until mature seed stage. Optimal S requirement was calculated according to the S needs of Capitol under a non-limiting condition from the study reported by Dubousset et al. [1]. Table S1 summarises the effects of the sulfur treatments on the mother plants. The seeds of each treatment, i.e., S400%, S70%, S20% and S5% seeds, were finally harvested at maturity and stored at 12 °C under vacuum in a desiccator until their use. For germination tests and investigations on seedling development, the mature seeds obtained for each treatment were sorted based on a diameter higher than 1.6 mm in order to exclude the few aborted seeds and the seeds fragments.

### 4.2. Germination Tests and Indexes

Fifty mature seeds per biological repetition (n = 3) were sown on Whatman filter paper soaked with 10 mL sterile water within Petri dishes (12 × 12 cm). The Petri dishes were then closed and placed in darkness inside a growth chamber at 20 °C and 70% relative humidity. Seeds that showed a completed radicle protrusion through the seed coat were counted as germinated. The germination percentage of the different seed lots was represented using Gompertz functions [14].

Five germination indexes were selected for this study. The germination capacity (%) was determined at 71 h. The time to reach 50% germination of the total number of seeds (T50) and 50% of the germination capacity (T’50) were calculated from the Gompertz functions. The speed of germination was calculated according to Bradbeer [15] using the following equation:

Speed of germination = ∑[(N_t_ − N_(t−1)_)/t]

where Nt is the percentage of germinated seeds observed at t hours.

The Coefficient of the Rate of Germination (CRG) was calculated according to Bewley and Black [3] with the following equation:

CRG = 100 × ∑N_t_/∑(t × N_t_).

### 4.3. Determination of Normal Seedling Rate

One hundred and fifty (150) mature seeds per biological repetition (n = 3) were germinated on perlite medium soaked with water for 14 days after sowing (14 DAS) with a cycle of 8 h dark (18 °C)/16 h light (25 °C) and a photon flux density of 400 μmol·m^−2^·s^−1^. Seedlings displaying at 14 DAS a radicle but no hypocotyl or a thick glassy radicle and/or hypocotyl were considered as abnormal. The percentage of seedlings with normal development is indicated for each S treatment in Table 2. The viable seedlings at 14 DAS were used for testing the sulphate uptake capacity of seedlings using a ^34^SO_4_^2−^ pulse method.

### 4.4. Protocol for Studying the Development and Sulphate Uptake Capacities of Seedlings Coming from Seeds Produced by Plants Supplied with Suboptimal Levels of Sulphate from the Beginning of the Plant Growth Cycle

Fourteen days after sowing on perlite soaked with water (14 DAS), normally developed seedlings obtained from each treatment (i.e., S400%, S70%, S20% and S5% seedlings) were fed with ^34^SO_4_^2−^ (pulse of 24 h) for testing their S uptake capacity. The ^34^SO_4_^2−^ was obtained by oxidation of 100 mg ^34^S-sulphur (Sigma Aldrich, St. Quentin Fallavier, France) with 10 mL of nitric acid (HNO_3_ at 65%) in a tube of mineralisation at 200 °C for 2 h as described in Salon et al. [33]. Seedlings were transferred for 24 h on perlite soaked with 25% Hoagland nutrient solution with 508.7 µM ^34^SO_4_^2−^ (5 atom% excess; 15 DAS + ^34^SO_4_^2−^) or under a S-restricted condition (8.7 µM SO_4_^2−^; 15 DAS-S). After this period, seedlings were collected and profusely rinsed with water for 1 min. Shoot and roots were separated, and, for 14 DAS seedlings, an aliquot of each was stored at −80 °C for RNA extraction and q-PCR analysis. For 14 and 15 DAS seedlings, the remaining root and shoot material was freeze-dried for the determination of dry weight, total S content, ^34^S labelling, sulphate and ^34^S-sulphate before (14 DAS) and after application of ^34^SO_4_^2−^ feeding (15 DAS + ^34^SO_4_^2−^) or under a S-restricted condition (15 DAS-S).

### 4.5. Biochemical and Molecular Analyses

#### 4.5.1. C, N, S and ^34^S Analysis

Freeze-dried samples were ground to a fine powder, weighed and prepared into tin capsules for determination of total C, N and S contents using an elemental analyser (EA3000, EuroVector, Milan, Italy) connected to a continuous flow isotope ratio mass spectrometer (IRMS; Isoprime, GV Instruments, Manchester, UK). The IRMS analysis provided the changes of the relative amount of ^34^S in excess in each sample derived from the tracer fed to the test plant.

#### 4.5.2. Determination of S-Sulphate Amount

Sulphate was extracted from 45 mg dry weight (DW) of seed samples ground to a fine powder, incubated twice with 2 mL of 50% ethanol at 40 °C for 1 h, centrifuged (10,000× *g*, 20 min), incubated twice with water at 95 °C for 1 h and centrifuged (10,000× *g*, 20 min). The supernatants were pooled and evaporated under vacuum (Concentrator Evaporator RC 10.22, Jouan, Saint-Herblain, France). The dry residue was resuspended in 2 mL of ultra-pure water. The sulphate concentration was directly determined by high performance liquid chromatography (HPLC, ICS3000, Dionex Corp., Sunnyvale, CA, USA).

#### 4.5.3. Determination of S-Protein Amount

For determination of S-protein amounts, soluble proteins were extracted by grinding 30 mg fresh weight of mature seeds in 0.5 mL of citrate Na-phosphate buffer (20 mM citrate and 160 mM Na_2_HPO_4_, pH 6.8). The homogenate was centrifuged at 12,000× *g*, 4 °C for 1 h and the resulting supernatant was used to determine the concentration in soluble proteins by protein-dye staining [34] using bovine serum albumin as standard. Proteins were then precipitated by adding four volumes of 10% TCA in acetone to one volume of extract. After storage at −20 °C overnight, the extract was centrifuged (12,000× *g*, 4 °C, 20 min) and the resulting pellet was washed twice with 1 mL 80% acetone and centrifuged (16,000× *g*, 4 °C, 3 min). Residual acetone was evaporated under vacuum at 45 °C, and the resulting pellet, resuspended in 0.1 mL of ultra-pure water, was placed into a tin capsule. The water was then evaporated under vacuum at 45 °C (Speedvac Concentrator 5301, Eppendorf, France), and dry protein extract was analysed for S content using an elemental analyser combined with an IRMS as described above for total S.

#### 4.5.4. Determination of Glutathione (GSH) Amounts by HPLC

Thiols were extracted by grinding 10 mg dry weight of seed in 0.2 mL of 0.1 M HCl. After centrifugation (20,000× *g*, 10 min), the supernatant was used to measure the content of total GSH after reduction with DTT by HPLC using the monobromobimane derivatisation method as described by Koprivova et al. [35].

### 4.6. Relative Expression of BnSultr1 and BnSultr4 Genes Using Q-PCR

Total RNA was extracted from 80 mg fresh weight of root or shoot samples at 14 DAS using the Mini Kit RNeasy from Qiagen (Qiagen, Courtaboeuf, France) according to the manufacturer’s protocol. Quantification of total RNA was performed by spectrophotometer at 260 nm (BioPhotometer, Eppendorf, Le Pecq, France) before Reverse Transcription (RT) and Quantitative PCR (Q-PCR) analyses. For RT, 1 µg of total RNA was converted to cDNA with an ‘iScript cDNA synthesis kit’ according to the manufacturer’s protocol (Bio-Rad, Marne-la-Coquette, France).

Q-PCR amplifications were performed using primers of *BnSultr1;1* (Accession No: AJ41640) and *BnSultr1;2* (Accession no: AJ311388) encoding transporters involved in sulphate uptake by roots, and primers of *BnSultr4;1* (Accession No: AJ416461) and *BnSultr4;2* (Accession No: FJ688133) encoding vacuolar sulphate transporters in *Brassica napus*. Q-PCR amplifications were performed by using *BnSultr4;1* forward primer: 5′-GACCAGACCCGTTAAGGTCA-3′ and reverse primer: 5′-TTGGAATCCATGTGAAGCAA-3′, *BnSultr4;2* forward primer: 5′-AGCAAGATCAGGGATTGTGG-3′ and reverse primer: 5′-TGCAACATTTGTGGGTGTCT-3′, *BnSultr1;1* forward primer: 5′-AGATATTGCGATCGGACCAG-3′ and reverse primer: 5′-GAAAACGCCAGCAAAGAAAG-3′, and *BnSultr1;2* forward primer: 5′-GGTGTAGTCGCTGGAATGGT-3′ and reverse primer: 5′-AACGGAGTGAGGAAGAGCAA-3′. *EF 1α* (Accession No: DQ312264) and 18S RNA (Accession No: X16077) were used as internal control genes and were amplified using EF1.1 forward primer: 5′-GCCTGGTATGGTTGTGACCT-3′ and reverse primer 5′-GAAGTTAGCAGCACCCTTGG-3′, and 18S forward primer: 5′-CGGATAACCGTAGTAATTCTAG-3′ and reverse primer 5′-GTACTCATTCCAATTACCAGAC-3′. Q-PCR reactions were performed with 4 μL of 200× diluted cDNA, 500 nM of primers and 1× SYBR Green PCR Master Mix (Bio-Rad, Marne-la-Coquette, France) in a ChromoFour System (Bio-Rad, Marne-la-Coquette, France). The specificity of PCR amplification was examined by monitoring the presence of the single peak in the melting curves after Q-PCR reactions. For each sample, the subsequent Q-PCR reactions were performed in duplicate, and the relative expression of the BnSultr transporters in each sample was compared to the control sample (corresponding to S400% seeds) and was determined with the ∆∆Ct method using the following equation:

Relative expression = 2 ^−(∆Ct sample − ∆Ct control)^

with

∆Ct = CtBnSultr − Ctreference genes

where Ct refers to the threshold cycle determined for each gene in the exponential phase of PCR amplification. Using this analysis method, relative expression of the *BnSultr* genes in the S400% seedlings samples was equal to one, by definition [36].

### 4.7. Statistics

The variability of the results is expressed by the average values for all biological replicates (n = 3 or 4) ± standard error (SE). The data were compared between treatments with the Mann–Whitney’s test, used for unpaired data, with a statistical significance fixed at *p* <0.05. The data were also compared between dates with the Wilcoxon test, used therefore for paired data, with a statistical significance fixed at *p* <0.05. These statistical tests were performed with Microsoft^®^ Excel 2011/XLSTAT©-Pro (Version 2013.3.01, Addinsoft, Inc., Brooklyn, NY, USA).

## Figures and Tables

**Table 1:** Seed dry weight and amounts of C, N, S, S-sulphate, S-protein and S-glutathione in mature *Brassica napus* seeds obtained from plants well-supplied with S and grown under S-limited conditions. Values correspond to means ± standard error (SE) (n = 3). Seeds (without fragments and aborted seeds) were obtained from plants subjected to plethoric S nutrition (400% of plant S needs provided, S400% seeds) or subjected to a S limitation from the beginning of plant growth with 70%, 20% and 5% of plant S needs provided (S70%, S20% and S5% seeds, respectively).

Seed Lot	Seed Dry Weight (mg·seed^−1^)	C Amount (mg·seed^−1^)	N Amount (µg·seed^−1^)	S Amount (µg·seed^−1^)	S-Sulphate Amount (µg·seed^−1^)	S-Protein Amount (µg·seed^−1^)	S-Glutathione Amount (ng·seed^−1^)
S400%	4.02 ± 0.2	2.62 ± 0.1	220.35 ± 13.61	58.95 ± 5.43	11.31 ± 1.76	7.21 ± 0.55	284.1 ± 28.2
S70%	4.27 ± 0.12	2.63 ± 0.07	235.24 ± 4.3	52.18 ± 3.06	9.19 ± 2.6	9.42 ± 0.49 *	231.5 ± 51.8
S20%	4.1 ± 0.25	2.55 ± 0.18	207.17 ± 16.89	18.2 ± 2.53 *	2.5 ± 0.41 *	6.26 ± 0.39	333.8 ± 46.6
S5%	3.99 ± 0.43	2.54 ± 0.3	209.4 ± 28.53	10.56 ± 2.25 *	0.91 ± 0.28 *	6.6 ± 0.64	161.9 ± 9.6 *

* Significant difference from the S400% seeds (*p* < 0.05).

**Table 2:** Germination indexes and normal seedling rate of *Brassica napus* seeds obtained from plants well S supplied and grown under S-limited conditions. Values correspond to means ± SE (n = 3). Seeds were obtained from plants subjected to plethoric S nutrition (400% of plant S needs provided, S400% seeds) or subjected to a S limitation from the beginning of plant growth with 70%, 20% and 5% of plant S needs provided (S70%, S20% and S5% seeds, respectively).

SEED Lot	Germination Capacity (%) ^a^	T50 (hours) ^b^	T’50 (hours) ^b^	Speed of Germination (hour^−1^) ^c^	CRG ^d^	Normal Seedling Establishment Rate (%) ^e^
S400%	100 ± 0.0	28.7 ± 1.0	28.7 ± 1.0	3.37 ± 0.1	2.03 ± 0.03	86.7 ± 1.8 °
S70%	100 ± 0.0	26.6 ± 0.9	26.6 ± 0.9	3.62 ± 0.11	2.1 ± 0.03	74.7 ± 2.3 *°
S20%	98 ± 1.2	24.6 ± 1.1	24.3 ± 1.2	3.91 ± 0.26	2.19 ± 0.05	66 ± 8.1 *°
S5%	75.3 ± 4.7 *	35.8 ± 1.2 *	31.6 ± 1.1	2.27 ± 0.13 *	1.96 ± 0.01	69 ± 2.8 *

^a^ Germination capacity determined at 71 h; ^b^ calculated from the Gompertz equation [14]; ^c^ as calculated by Bradbeer [15]; ^d^ Coefficient of the Rate of Germination (CRG) as calculated by Bewley and Black [3]; ^e^ determined at 14 days after sowing (DAS). * Significant difference from the value obtained for the S400% seeds (*p* < 0.05). ° Significant difference between germination capacity and normal seedling rate (*p* < 0.05).

## Supplementary Materials

The following are available online at http://www.mdpi.com/2223-7747/8/1/12/s1, Figure S1: S amount (a) and S repartition (b) of *Brassica napus* cv. Yudal green seedlings, 14 days after sowing on water (14 DAS) and after 24 h treatment of 14 DAS green seedlings with 508.7 µM ^34^SO_4_^2−^ at 5 atom% excess (15 DAS + ^34^SO_4_^2−^) and with 8.7 µM SO_4_^2−^ (15 DAS-S), Figure S2: Differences in ^34^S amount in excess in roots and shoots of oilseed rape green seedlings (cv. Yudal) between 14 DAS and 15 DAS-S and between 14 DAS and 15 DAS + ^34^SO_4_^2−^, Table S1: Plant dry weight, seed yield, number of seeds per plant and seed dry weight at harvest for plants well-supplied with S and grown under S-limited conditions. Values correspond to means ± SE (n = 3). Plants were either grown under plethoric S nutrition (400% of plant S needs provided, S400% seeds) or subjected to a S limitation from the beginning of plant growth with 70%, 20% and 5% of plant S needs provided (S70%, S20% and S5% seeds, respectively). Seed yield was determined without any sorting. The slight discrepancy observed between the calculation of seed yield from ‘seed dry weight’ × ‘number of seeds per plant’ and the reported values of seed yield is due to the manual removal of some seed fragments when seeds were counted and weighted. * Significant difference from the value of S400% (*p* < 0.05).
