# Supplementary material for: Germinative and Post-Germinative Behaviours of Brassica napus Seeds Are Impacted by the Severity of S Limitation Applied to the Parent Plants
Source: Plants (Basel). 2019 Jan 5;8(1):12. doi: 10.3390/plants8010012 (PMC6359240; doi:10.3390/plants8010012)
Supplement: Supplementary file 1 [file plants-08-00012-s001.pdf]

Supplementary Materials:

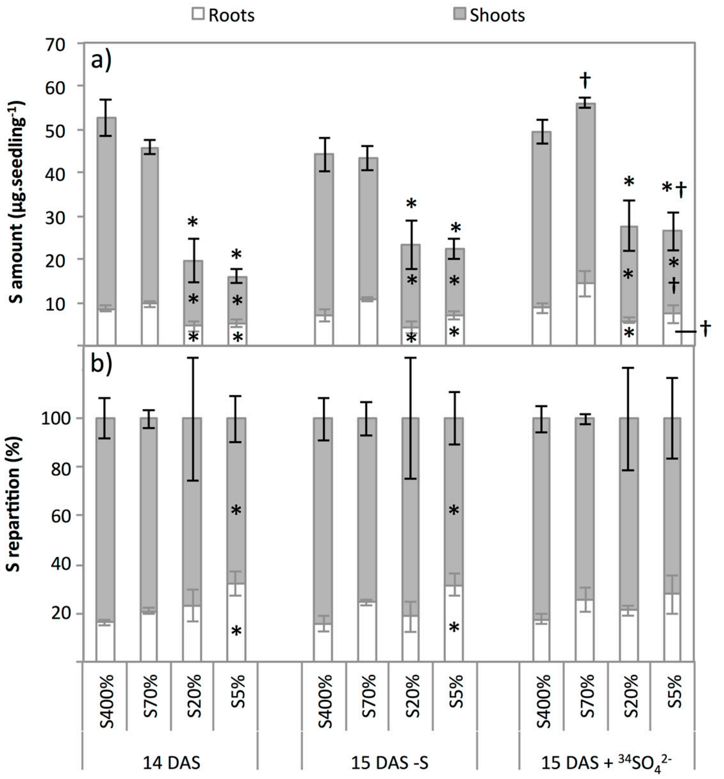

Figure S1: S amount (a) and S repartition (b) of *Brassica napus* cv. Yudal green seedlings, 14 days after sowing on water (14 DAS) and after 24 h treatment of 14 DAS green seedlings with 508.7 µM $^{34}SO_4^{2-}$ at 5 atom% excess (15 DAS + $^{34}SO_4^{2-}$) and with 8.7 µM SO_4^{2-} (15 DAS -S). * Significant difference from the value of S400% green seedlings ($p < 0.05$). † Significant difference from the corresponding value at 14 DAS ($p < 0.05$). Signs (* and/or †) on the top of the bars are for the whole plant (shoot and root) while signs inside the bar are for root or shoot specifically.

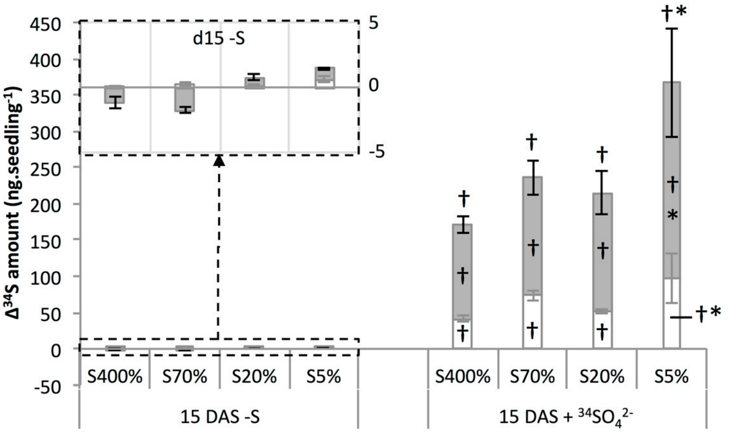

Figure S2: Differences in ^{34}S amount in excess in roots and shoots of oilseed rape green seedlings (cv. Yudal) between 14 and 15 DAS -S and between 14 and 15 DAS +$^{34}SO_4^{2-}$. * Significant difference from the value of S400% green seedlings ($p < 0.05$). † Significant difference from the corresponding value with 15 DAS -S ($p < 0.05$). Signs (* and/or †) on the top of the bars are for the whole plant (shoot and root) while signs inside the bar are for root or shoot specifically.

Table S1: Plant dry weight, seed yield, number of seeds per plant and seed dry weight at harvest for plants well supplied with S and grown under S-limited conditions. Values correspond to means ± SE (n = 3). Plants were either grown under plethoric S nutrition (400% of plant S needs provided, S400% seeds) or subjected to a S limitation from the beginning of plant growth with 70%, 20% and 5% of plant S needs provided (S70%, S20% and S5% seeds respectively). Seed yield was determined without any sorting. The slight discrepancy observed between the calculation of seed yield from 'seed dry weight' x 'number of seeds per plant' and the reported values of seed yield is due to the manual removal of some seed fragments when seeds were counted and weighted. * Significant difference from the value of S400% ($p < 0.05$).

	Plant dry weight ($gDW·plant^{-1}$)	Seed yield ($gDW·plant^{-1}$)	Number of seeds per plant	Seed dry weight ($mg·seed^{-1}$)
S400%	25.37 ± 0.69	3.82 ± 0.63	1034 ± 220	3.95 ± 0.24
S70%	23.53 ± 0.86	3.53 ± 0.49	893 ± 173	4.12 ± 0.07
S20%	20.35 ± 1.2	3.76 ± 0.55	967 ± 189	4.01 ± 0.25
S5%	16.34 ± 1.32*	1.13 ± 0.16*	304 ± 58*	3.85 ± 0.43
